# Weighted Gene Co-Expression Network Analysis Reveals Hub Genes Contributing to Fuzz Development in *Gossypium arboreum*

**DOI:** 10.3390/genes12050753

**Published:** 2021-05-17

**Authors:** Xiaoxu Feng, Shang Liu, Hailiang Cheng, Dongyun Zuo, Youping Zhang, Qiaolian Wang, Limin Lv, Guoli Song

**Affiliations:** 1 State Key Laboratory of Cotton Biology, Institute of Cotton Research, Chinese Academy of Agricultural Sciences, Anyang 455000, China; bbxe2013@163.com (X.F.); lsxbdz@163.com (S.L.); pser2010@163.com (H.C.); zdy041@163.com (D.Z.); zyp547550790@163.com (Y.Z.); wangqiaolian@caas.cn (Q.W.); llm0372@126.com (L.L.); 2 Plant Genetics, Gembloux Agro Bio-Tech, University of Liège, 5030 Gembloux, Belgium

**Keywords:** transcriptome analysis, WGCNA, fuzz, module, hub genes

## Abstract

Fuzzless mutants are ideal materials to decipher the regulatory network and mechanism underlying fuzz initiation and formation. In this study, we utilized two *Gossypium arboreum* accessions differing in fuzz characteristics to explore expression pattern differences and discriminate genes involved in fuzz development using RNA sequencing. Gene ontology (GO) analysis was conducted and found that DEGs were mainly enriched in the regulation of transcription, metabolic processes and oxidation–reduction-related processes. Weighted gene co-expression network analysis discerned the MEmagenta module highly associated with a fuzz/fuzzless trait, which included a total of 50 hub genes differentially expressed between two materials. *GaFZ*, which negatively regulates trichome and fuzz formation, was found involved in MEmagenta cluster1. In addition, twenty-eight hub genes in MEmagenta cluster1 were significantly up-regulated and expressed in fuzzless mutant DPL972. It is noteworthy that Ga04G1219 and Ga04G1240, which, respectively, encode Fasciclin-like arabinogalactan protein 18(FLA18) and transport protein, showed remarkable differences of expression level and implied that they may be involved in protein glycosylation to regulate fuzz formation and development. This module and hub genes identified in this study will provide new insights on fiber and fuzz formation and be useful for the molecular design breeding of cotton genetic improvement.

## 1. Introduction

Trichomes are highly specialized cells originating and extending from the epidermal surface of leaves, stems, petal bases and seeds in plants [1,2]. They contribute to a variety of unessential but important functions for plant growth, including protections against pathogens, insects or even herbivores, water regulation and increasing the tolerance to extreme high temperature and UV irradiation [3,4]. Trichomes could be characterized and classified into various types by their morphology and nature, e.g., uni-/multi-cellular, non-/branched or non-/glandular [3,5,6]. Numerous molecular genetic studies have provided much insight into the molecular genetic mechanisms and regulatory networks associated with trichome formation. Plenty of functional genes were identified to regulate the mode of epidermal cell development in Arabidopsis, rice, maize, tomato, *Artemisia annua*, such as MYB-bHLH-WD40(MBW) complex [7,8], *OsHL6* [9], *OsWOX3* [10], *ZmOCL4* [11], *ZmSPL10/14/26* [12], *SlMYC1* [13], *SlWo* [14], *AaHD1* [15].

Cotton fibers are single-celled and unbranched trichomes that outgrow from the seed epidermal cells and successively undergo a highly polarized elongation similar to leaf trichomes [16,17]. Therefore, cell fate determination and elongation patterns unveiled from Arabidopsis trichomes may provide a useful framework involved in cotton fiber initiation and elongation. It has been reported that the orthologue members in the Arabidopsis MBW complex also evolved crucial roles in cotton fiber formation [18]. Besides, recent studies have uncovered various genes involved in multiple pathways to govern fiber initiation and development. *GhPIN3a* [19,20], *GhARF2/18* [21] and *GhHD1* [22,23] were reported as crucial members to regulate fiber initiation in cotton by responding to various phytohormones. Furthermore, *GaDEL65* was predominantly associated with fuzz initiation and may interact with *GhMYB2/3* and *GhTTG3* to regulate cotton fiber elongation [24]. According to previous surveys, *GhPEL76* [25], *GhSK13* [26], *GhAP2L* [26], *GhHDZ5* [26] and *Gh_DNF_YB19* [27], have been identified to extensively regulate fiber elongation responsive to the phytohormone signaling pathway, respectively. *AKR2A* modulates the biosynthesis pathway of very long-chain fatty acids and participates in fiber elongation regulation [28]. More recently, a set of transcription factors, such as *GhFSN1* [29], *GhHOX3* [30] and the miR319-targeted *GhTCP4* [31], were identified to remarkably playing positive or negative roles in the second cell wall deposition stage.

Fiber initiation stage determines the final fiber yield. Hence it is essential and necessary to highly explore the orchestrating network governing fiber cell differentiation and fiber formation [32]. According to the initiating time and final length, cotton fibers can be divided into lint and fuzz. Though most research studies focused on fiber initiation and elongation, the essential functional genes and the mechanisms underlying fuzz initiation and development remain largely unknown.

Fiber mutant and natural species exhibit diverse or significant fiber characteristics and are great resources to decipher the mechanisms underlying fiber and fuzz formation. With the release of high-quality sequencing data from multiple cotton species, bioinformatics-based analyses have also facilitated functional gene mapping and network regulation exploration. *GhMML3* and *GhMML4* have been proposed to be responsible for the fuzzless or fiberless phenotypes in tetraploid fiber mutant lines [32,33,34,35,36,37]. Compared with tetraploid cotton, diploid *G. arboreum*, a relatively simple genome, is potentially highly suitable for digging candidate genes corresponding to mutant traits. It has been reported that transcript splicing mistakes occurred because of a critical point mutation in *GaHD1* in a recessive fiber mutant *sma-4(ha)* and further affects fiber and trichome initiation and retards elongation by cellular H_2_O_2_ and Ca^2+^ signals [38]. Meanwhile, through whole genome re-sequencing and genetic linkage analyses, Liu et al. proposed *GaGIR1* and *GaMYB25-like* as crucial candidate genes controlling fuzz development and established *GaHD-1* to be the candidate gene responsible for the lintless trait in seven fuzzless accessions and one lintless accession of *G. arboreum* [39]. 

In a previous study, we fine-mapped a dominant fuzzless gene to a 70-kb region and speculated *GaGIR1* (GLABRA2-interacting repressor, Ga08G0121) as the likely candidate [40]. Recently, based on a genome-wide association study(GWAS), a 6.2 kb insertion, larINDEL_FZ_, located ~18 kb upstream of *GaFZ* (Ga08G0121) has been identified to be an enhancer to elevate the expression level of *GaFZ* and negatively regulate fuzz and trichome formation [41]. Although much progress has been achieved, the mechanism and regulation network remains mysterious and elusive. 

In this study, we utilized RNA-Seq for screening genes related to fuzz initiation development in two *G. arboreum* accessions and validated our data with qPCR. We also performed weighted co-expression gene network analysis to identify the modules and hub genes co-expressing with *GaFZ* and contributing to the fuzz development. Our results provide valuable gene resources and novel insights into mechanisms and regulatory networks of fuzz-associated genes.

## 2. Materials and Methods

### 2.1. Plant Materials

Plants of *G. arboreum* DPL971 with normal fuzz and lint fibers and its isogenic fuzzless mutant line DPL972 were grown under standard agronomic field conditions at the Institute of Cotton Research, Chinese Academy of Agricultural Sciences (CAAS), Anyang (E 114°48′, N 36°06′), China. All lines were strictly self-pollinated. Flowers were firstly tagged at 0 DPA, and cotton bolls were collected in morning (9:00–11:00) at 1, 3 and 5 DPA, respectively; the ovules were dissected and frozen in liquid nitrogen for RNA extraction. 

### 2.2. RNA Extraction and RNA-Seq

All samples were finely ground before RNA isolation. Total RNA was extracted following the manufacturer’s instructions (TIANGEN BIOTECH Beijing, China). Two biological replicates from each sample were used for this experiment. The quantity and quality of total RNA were monitored by NanoDrop 2000 spectrophotometer and 1% agarose gel electrophoresis. RNA samples of good quality were used for subsequent deep sequencing and quantitative real-time PCR (qRT-PCR) analysis, and cDNA libraries were constructed and subjected to 101-cycle paired-end sequencing on an Illumina HiSeq4000 platform at Berry Genomics (Beijing, China). All raw data were deposited to the NCBI’s Sequence Read Archive (SRA) and available with the accession number PRJNA729876.

### 2.3. RNA-Seq Data Analysis

Raw sequences obtained from the 12 sample libraries were assessed and filtered by Fastp (version = 0.20.1). The GC content and Q30 were calculated by FastQC. Hisat2 (version = 2.2.1) was used to align the remaining clean data to reference genome of *G. arboreum* [42]. The number of fragments per kilobase of transcript per million mapped reads (FPKM) of each transcript in the fuzzless mutant DPL972 and wild-type DPL971 was calculated using StringTie (version = 2.1.1) software. Based on read counts obtained from StringTie, differentially expressed genes (DEGs) between the mutant and the wild type were identified by using the DESeq2 with false discovery rate (FDR) < 0.05 and |log2 (ratio)| ≥ 1. The KMeans-clustering method was used for clustering DEGs. KMeans method was performed by python package Sklearn (version = 0.19.2). Gene Ontology (GO) and Kyoto Encyclopedia of Genes and Genomes (KEGG) were conducted via Cotton Functional Genomics Database (https://cottonfgd.org/, accessed on 9 March 2021) to determine the main biological functions and pathways of the significant DEGs.

### 2.4. qRT-PCR

To verify the reproducibility and authenticity of RNA-Seq, qRT-PCR was also performed in this study. The extracted total RNA samples with 1 µg concentration were used as template to reverse transcription using TransScript All-in-one First-strand cDNA Synthesis SuperMix (TransGen Biotech, Beijing, China) according to the manufacturer’s instructions. QRT-PCR amplifications were conducted using TransStart TOP Green qPCR SuperMix (TransGen Biotech, China) on an ABI QuantStudio5 Real-time PCR System (Applied Biosystems, Foster City, CA, USA). DEG expressions were normalized using *GaHis3* as reference gene. A total of eleven pairs of specific primers were designed from with NCBI Primer-BLAST (http://www.ncbi.nlm.nih.gov/tools/primer-blast/, accessed on 9 March 2021) and qPCR Primer Database (https://biodb.swu.edu.cn/qprimerdb/, accessed on 9 March 2021). Primer sequences were provided in Supplementary Table S1. Each qRT-PCR reaction was performed in triplicate and relative expression levels were calculated using the 2^−^^∆∆ct^ method.

### 2.5. Construction of Co-Expression Network and Network Analyses

To construct the co-expression network underlying two accessions, transcripts with FPKM count ≥ 5 count were used and all the outliers were removed. The WGCNA (weighted gene co-expression network analysis) R package was implemented to analyze co-expression networks and select hub genes which highly correlated with fuzz characteristic [43,44,45]. The dendrogram was generated using the cutreeDynamicTree algorithm, with a limits of minimum module size of 30 genes. A weighted correlation threshold of ≥0.85 was set to screen modules correlated with fuzz/fuzzless traits. The Sankey diagram was performed by Pyecharts (version = 1.9.0). The interaction networks among genes in selected modules were constructed using Cytoscape and visualized by Pyecharts. The membership (KME) values threshold of ≥0.80 was set to screen the module hub genes. Heatmap for visualization of expression was generated by python package Seaborn (version = 0.9.0).

## 3. Results

### 3.1. Data Processing and Analysis 

To capture a global view and the temporal gene expression changes during fiber and fuzz initiation development, a transcriptome profiling experiment was performed from 12 cotton ovule libraries including wild type DPL971 and the fuzzless mutant DPL972 during the fuzz and fiber initiation stage (+1DPA, +3DPA and +5 DPA). Two biological replicates were adopted for each material in each time point. After removing adaptor and low-quality reads, a total of 90.27 Gb of cleaning data was generated, including 30.1 million reads, averaging 7.52 G and 25,077,291 reads per sample (Supplementary Table S2). For all samples, the GC content varied from 44% to 45%, and Q30 altered from 95.24% to 95.51%. All clean reads were aligned to the newly published reference *G. arboreum* genome, and 89.00% to 93.02% of the sequenced reads from each library could be perfectly mapped. These results indicated that our transcriptome sequencing data were of high quality and good enough to further identify DEGs and construct the regulatory network.

### 3.2. Differential Gene Expression in DPL971 and DPL972 during Fuzz Initiation

**Figure 1:**
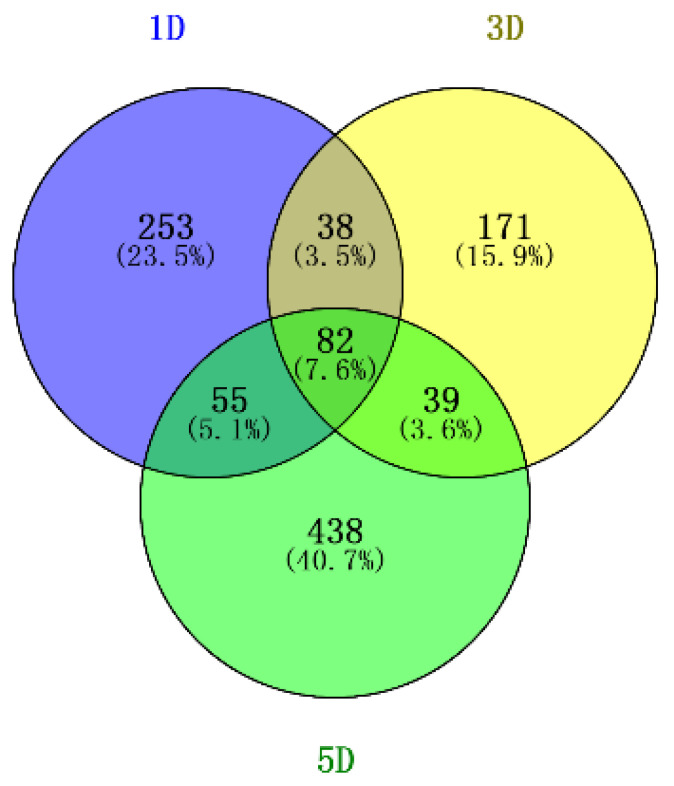
Venn diagram of DEGs identified from two diploid cotton accessions; 1D, 3D, 5D represents 1 Days post-anthesis (DPA), 3 DPA, 5 DPA.

A total of 1076 DEGs were detected using the criteria of false discovery rate (FDR) ≤0.05 and |log2 Ratio| ≥ 1. The number of DEGs between the WT and mutant during fuzz initiation stages (+1DPA, +3 DPA and +5 DPA) was 428, 330 and 614, respectively (Figure 1). Besides, eighty-two genes shared overlapping and differentially co-expression across the three different time points, suggesting that the transcriptome variation between the two accessions maintains a stable state through fuzz determination and the formation stage. 

**Figure 2:**
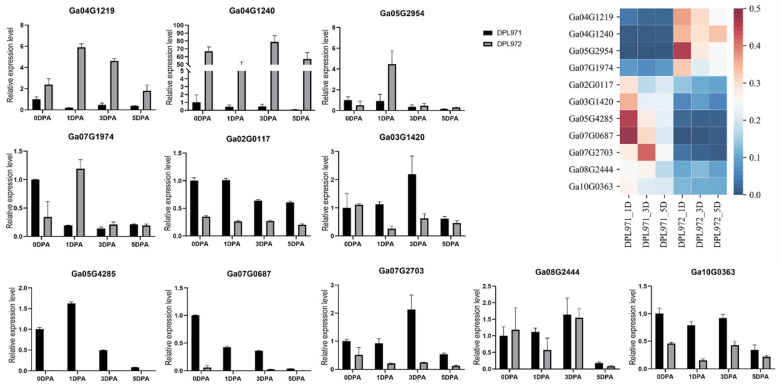
Validation of DEGs identified from transcriptome analysis with qRT-PCR. The heatmap on the top right of figure represents the relative transcription abundances (based on FPKM).

To verify the reproducibility and authenticity of the RNA-seq data, qRT-PCR was performed on selected 11 DEGs with gene-specific primers (Supplementary Table S2). As shown in Figure 2, all 11 genes showed a consistent expression pattern between the RNA-seq data and the qRT-PCR results. Genes significantly up-regulated in RNA-Seq data also exhibited an up-regulation in qPCR, and vice versa. These results also supported the reproducibility and authenticity of transcriptomic data, which were utilized in downstream co-expression network and functional enrichment analysis.

### 3.3. Functional Enrichment of DEGs

**Figure 3:**
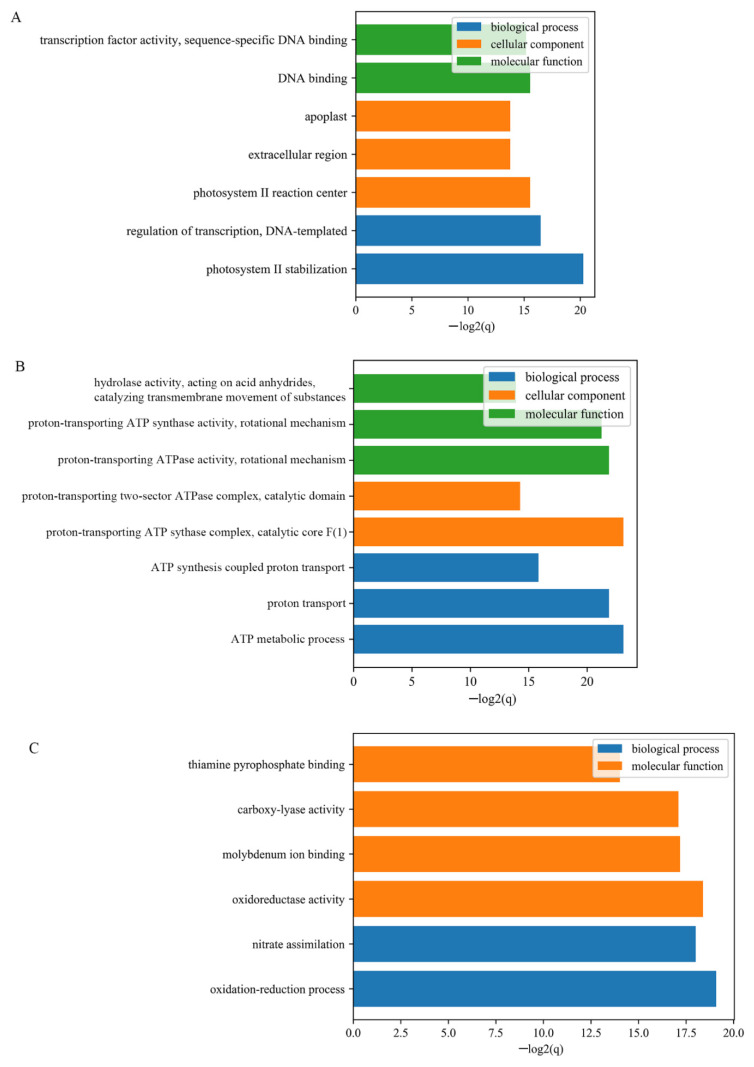
GO enrichment analysis of DEGs at three fiber developmental stages; (**A**), DEGs detected from 1DPA; (**B**), DEGs detected from 1DPA; (**C**) DEGs detected from 1DPA.

To estimate the main biological functions and regulations of identified DEGs, Gene ontology enrichment analysis and KEGG were performed on gene IDs that involved in each developmental points. As shown in Figure 3, DEGs generated from 1D were enriched in the following Molecular Function terms “transcription factor activity, sequence-specific DNA-binding” and “DNA-binding”. They were also enriched in terms of “photosystem II stabilization” and “regulation of transcription, DNA-templated” from the biological process category. The enrichment of cellular component annotations included the terms of photosystem II reaction and extracellular region. While DEGs identified from 3D were mainly enriched in ATP synthesis and metabolic process and proton transporting associated terms, and DEGs identified from 5D were clustered into terms of “oxidoreductase activity” and “oxidation-reduction process”. Further, the pathway enrichment analysis revealed that cell cycle, oxidative phosphorylation, biosynthesis of secondary metabolites, and glycolysis/gluconeogenesis were enriched among the DEGs and might be involved in fuzz initiation in cotton epidermal cell differentiation and development (Supplementary Table S3).

### 3.4. Co-Expression Network Construction and Module Mining

**Figure 4:**
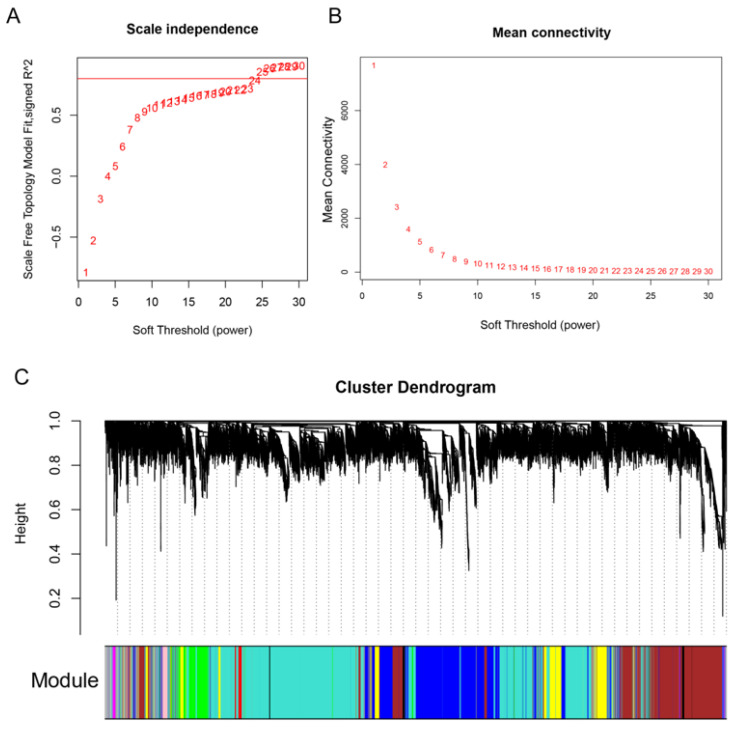
Module identification by weighted gene co-expression network analysis (WGCNA). (**A**,**B**) represent the soft threshold with scale independence and mean connectivity. (**C**), Hierarchical dendrogram reveals co-expression modules identified by WGCNA. Each leaf represents one gene. Ten modules were identified based on calculation of eigengenes; each module was decorated with a different color.

To uncover important genes and regulation pathways involved in fuzz and fiber initiation stage, we performed the R WGCNA package on all 12 samples. A soft-threshold power of 25 was introduced into the network topology to reveal the scale independence and mean connectivity of the network (Figure 4A,B). Based on the fragments per kilobase per million mapped (FPKM) expression matrix and phenotypic traits including fuzz or fuzzless characteristics and three developmental points, a total of 20,601 genes in 12 samples were clustered and divided into 10 modules which decorated with diacritical colors (Figure 4C). The outliers and genes whose FPKM values <5 in all samples were excluded in this analysis.

**Figure 5:**
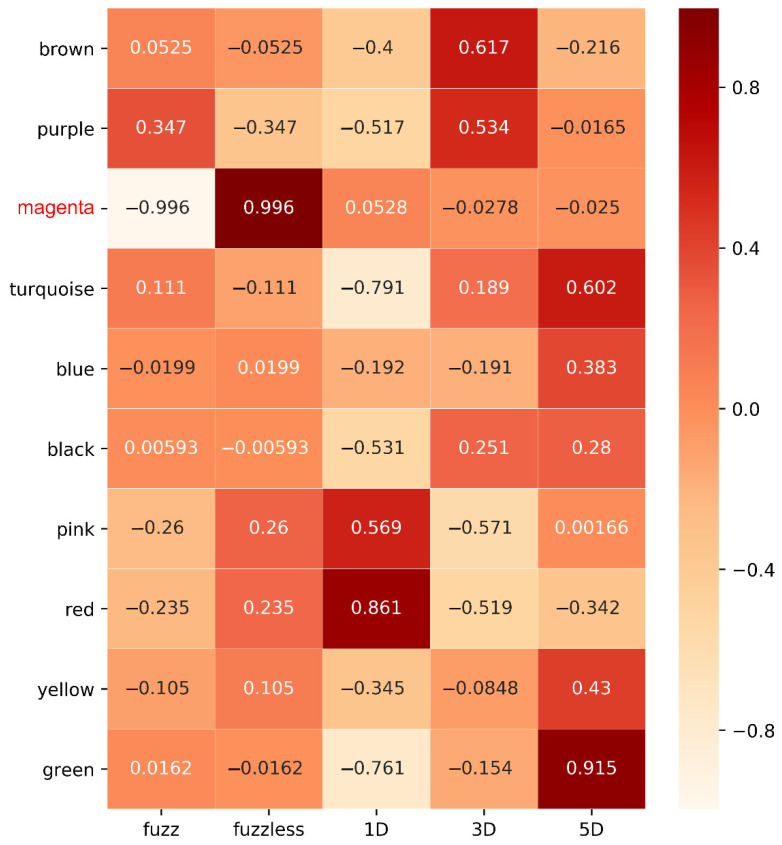
The WGCNA showed the MEmagenta module is significantly associated with fuzz formation. Each row means a module, and the correlation coefficient are shown in each square and the *p*-value was list in Table S4. The names in red in left represent the module highly associated with fuzz development.

**Figure 6:**
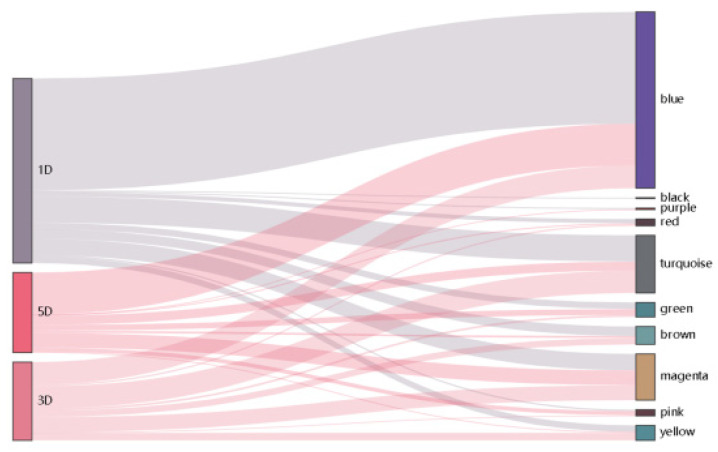
The Sankey diagram represents the distributions of DEGs in each module.

Heatmap was graphed to unveil the correlation between modules and traits, *p*-values were presented in Supplementary Table S4. After calculating the correlation coefficient, one module was strongly associated with fuzz and fuzzless characteristics, indicating it might be highly contributed to fuzz initiation and formation (Figure 5). The MEmagenta module exhibited extremely exclusive correlations with fuzz/fuzzless traits (*r* = 0.996, *p*-value = 6.27 × 10^−12^). This module was analyzed further. In addition, we also identified red, brown and green modules significantly and substantially correlated with fiber development of 1, 3 and 5 DPA, respectively, suggesting genes involved in these modules most probably participated in fiber initiation and elongation during fiber developmental stages. Besides, the Sankey diagram was generated to detect the distribution of DEGs in each module, revealing that DEGs from MEmagenta distributed evenly at 1, 3, 5 DPA (Figure 6). 

### 3.5. Functional Enrichment and Expression Analysis of Fuzz-Associated Hub Genes

To obtain more key genes involving the process of fuzz initiation, we did further analysis on the MEmagenta module which comprises 98 genes. To better understand gene functions and regulatory networks, GO and KEGG enrichment analyses were also used to analyze the fuzz-correlated module. The top terms were, respectively, presented in Supplementary Table S5 and Supplementary Figure S1. The GO results demonstrate genes in the MEmagenta module were enriched in transferase activity and transport protein, and the KEGG revealed genes in this module were best matched with the NADH dehydrogenase (ubiquinone) 1 α sub-complex item. 

**Figure 7:**
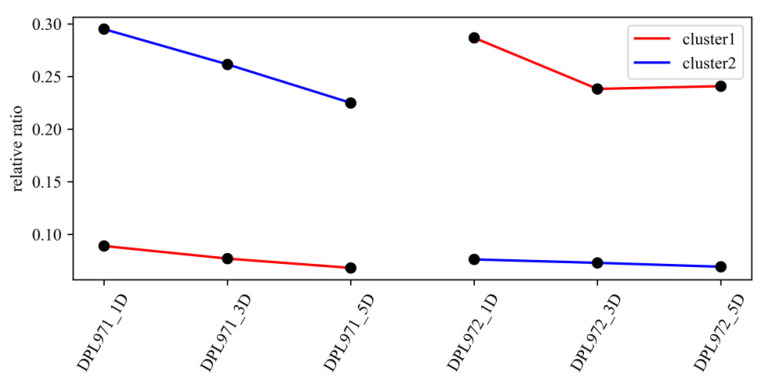
K-means clustering of DEGs in the MEmagenta module.

According to different expression profiles, but similar functions, genes in the MEmagenta module could be divided into two clusters using the K-means clustering method (Figure 7). Based on the kME value, the intramodular connectivity ranks and genes with kME value >0.8 were selected as hub genes that would determine the network centrality and potential crucial roles in fuzz/fuzzless characteristics. These hub genes were listed in Supplementary Table S6 and complex interaction networks in the co-expression module were respectively shown in Supplementary Figure S2 to highlight the potential networks underlying fuzz development.

**Figure 8:**
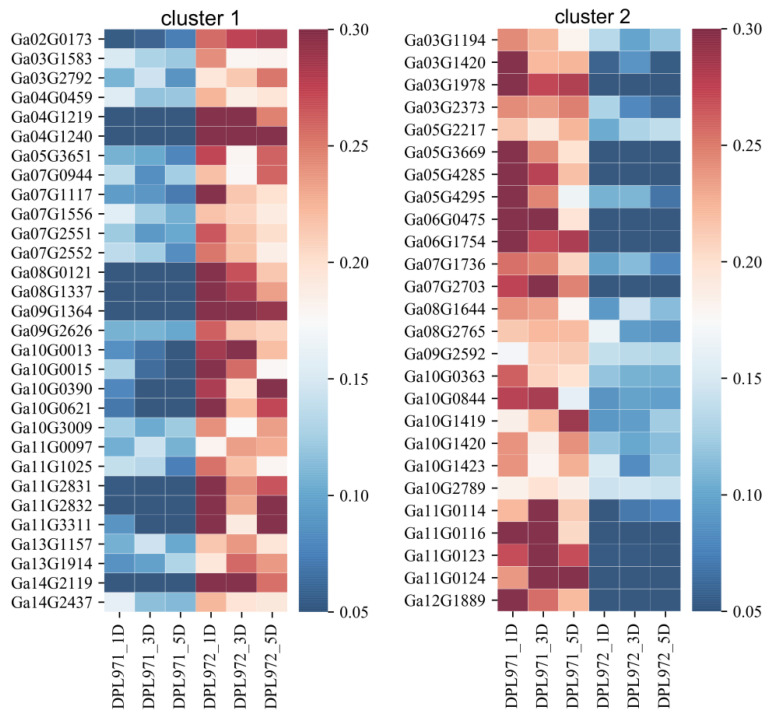
The expression heatmaps of hub genes in the MEmagenta module. The right side of figure represents the relative transcription abundances (based on FPKM). The gene IDs were listed on the left and sample names were displayed on the bottom.

To uncover the expression patterns of hub genes, we compared DEGs and genes in the selected module. Interestingly, a total of 50 out of 56 identified hub genes exhibited highly differential expression levels between DPL971 and DPL972 (Figure 8), indicating these hub genes play important roles in regulating fuzz initiation and development. In addition, previous studies reported *GaFZ* (Ga08G0121) differentially expressed between DPL971 and DPL972 due to a large insertion and thus negatively regulates fuzz formation [40,41]. The gene was also identified and involved in MEmagenta module cluster1, which we selected as main cluster negatively correlating with fuzz trait (positively correlating with fuzzless trait), suggesting the reliability of the interested module and network identified from WGCNA. What is more, similar to *GaFZ*, Ga04G1219 encoding Fasciclin-like arabinogalactan protein 18(FLA18) and Ga04G1240 encoding transport protein exhibited dramatic up-regulated expression in fuzzless DPL972 compared to DPL971 (Figure 2). It suggested that they and their interaction network may be involved in fuzz formation and development via controlling protein-glycosylation and responding to phytohormones.

## 4. Discussion

In the present study, we investigated gene expressions and regulatory networks in two *G. arboreum* accessions with a significant difference in fuzz characteristic. By performing transcriptomic analysis of fiber-attached ovules collected from 1, 3 and 5 DPA, DEGs were identified, respectively. GO enrichment analysis presented that DEGs in 1 DPA were mainly enriched in transcription factor activity, and DEGs in 3 and 5 DPA were clustered into items of ATP synthesis and proton transporting, oxidoreductase activity, respectively. Further, we established a weighted gene co-expression network analysis underlying fuzz initiation and formation. WGCNA revealed that only one co-expression module, MEmagenta, had a dominantly high correlation with fuzz trait, and the genes in these modules were significantly enriched in items of transferase and transport protein based on the GO enrichment analysis. 

### 4.1. Hub Genes in MEgrey60 Cluster Might Be Involved in Regulatory Network Responsible to Fuzz Initiation

Fiber and fuzz initiation undergo rather complex and obscure processes. Until now, multiple loci and factors have been identified to control or regulate fiber and fuzz initiation. In previous studies, larINDEL_FZ_ was screened out to be responsible for the fuzzless phenotype in various diploid accessions through genome wide association study (GWAS) and enhanced the expression level of *GaFZ* [41]. Overexpression of *GaFZ* in tetraploid cotton presented a reduction of trichomes and fuzz, suggesting GaFZ might be contributed to fuzz initiation and formation. In our study, *GaFZ* significantly differentially expressed in two accessions and was involved in MEmagenta module cluster1with a correlation of 0.816. By conducting the prediction and comparison of protein structure, we found this protein shared a high similarity with DNA-directed RNA polymerase II subunit RPB12, which functions as part of activated transcription complex (Supplementary Figure S2). It implies *GaFZ* might act in this way to regulate other genes to affect fuzz initiation. Besides, a comparison of the results of WGCNA and DEGs analysis indicated that 93 out of 98 genes in the MEmagenta module exhibited differential expression levels. In addition, 50 out of 56 hub genes, which were identified as critical genes to be significantly correlated with fuzz initiation and formation, presented notably differential expression patterns. In previous studies, glycosyltransferase31 could influence fiber initiation and elongation via controlling the glycosylation of FLAs and regulating pectin biosynthesis [46,47,48,49,50,51,52,53]. Interestingly, genes encoding FLA, UGT, RLK, transport protein were also identified in hub genes in MEmagenta cluster1. Together with GaFZ, these differentially expressed hub genes may be the key regulators underlying fuzz initiation and development in G. arboreum by protein glycosylation and phytohormone response. These results also indicated that the combination of transcriptomic analysis and WGCNA is highly efficient and superior in detecting and discerning functionally associated genes as well as gene regulatory networks. 

### 4.2. Multiple Protein Kinase Genes may Regulate Fuzz and Plant Development in G. arboreum

Cotton is a great model for detecting the mechanism of cell differentiation and elongation. Understanding the molecular pathway regulating fiber and fuzz initiation can provide essential and valuable information for improving the fiber production and prompting the process of the molecular design breeding. Various protein kinases are important and essential in response to biotic and abiotic stresses as well as regulating plant and fiber development [54,55,56,57,58,59]. Mitogen-activated protein kinase (MAPK) cascades have been reported to alter pathogen resistance through gibberellins (GA) signaling pathway and participate in cotton fiber elongation by phosphorylating its downstream proteins [60,61]. Ectopic expression of receptor-like kinases (RLKs) improved cotton tolerance and resistance to fungal pathogen infections [62,63,64,65,66]. Calcineurin B-like protein and CBL-interacting protein kinases (CIPKs) were proved to be associated with cotton oil and sugar content and stress responses [59,67,68,69,70]. Glycosyltransferases (UGT) were identified to participate and function in biosynthesis process of hemicellulose glucuronoxylan xylan during fiber and plant development whereas xyloglucan endo-transglycosylases /hydrolases (XTH) would limit the elongation of cotton fiber [56,71,72]. Glutathione S-transferase genes had an important role in resistance of Verticillium wilt, and sulfotransferases (SOTs) genes might be involved in cotton fiber development [73,74]. In the present study, CBL-interacting protein kinase 9 (CIPK9), UDP-glycosyltransferase 83A1 (UGT83A1), MDIS1-interacting receptor-like kinase 2 (MIK2), probable inactive histone-lysine N-methyltransferase (SUVR1) and Inositol-tetrakisphosphate 1-kinase 2 (ITPK2) were identified as hub genes in the MEmagenta module, indicating that these protein kinase-related genes might be crucial and potential targets for the regulation of fiber/fuzz initiation and development in *G. arboreum*, and they are well worth further and deeper understanding and research.

## 5. Conclusions

In this study, we collected the fiber-attached ovules samples at three fiber developmental stages (1, 3 and 5 DPA) for RNA-Seq from a pair of *G. arboreum* accessions with different fuzz traits, and through transcriptome comparisons, 428 DEGs at 1DPA, 330 DEGs at 3 DPA and 614 DEGs at 5 DPA were identified, respectively. WGCNA identified a MEmagenta module highly associated with fuzz/fuzzless development. Within this module, a total of 93 genes exhibited significantly differential expression patterns between two accessions. *GaFZ,* which has been reported to negatively regulate trichome and fuzz formation, was found involved in MEmagenta cluster1. Besides, the expression levels of twenty-eight hub genes in MEmagenta cluster1 were notably up-regulated and twenty-two hub genes in cluster2 were dominantly down-regulated in fuzzless mutant DPL972. Furthermore, the coding genes of FLA18, UGT and transport protein showed remarkable differences of expression profiles and implied that they may be involved in protein glycosylation to regulate fuzz development. This study thus unveils the regulatory mechanisms underlying fuzz initiation and the development stage in *G. arboreum*, and may facilitate the molecular design breeding of cotton with improved fiber characteristics.

## Data Availability

The data presented in this study are available in the article and supplementary material.

## Supplementary Materials

The following are available online at https://www.mdpi.com/article/10.3390/genes12050753/s1, Figure S1: KEGG enrichment analysis of genes in the MEmagenta module; Figure S2: Diagram of interaction networks of genes in the MEmagenta module; Figure S3: Comparison of protein structures between GaFZ and RPB12, Table S1: Primers used in qRT-PCR, Table S2: Statistics and alignments of RNA-seq reads mapped to reference genome, Table S3: KEGG enrichment analysis of DEGs obtained from two accessions, Table S4: *p*-values of relationships between module and sample, Table S5: GO enrichment analysis of hub genes in the MEmagenta module, Table S6: Hub genes in the MEmagenta module.
