# Supplementary figures and images for: Weighted Gene Co-Expression Network Analysis Reveals Hub Genes Contributing to Fuzz Development in Gossypium arboreum
Source: Genes (Basel). 2021 May 17;12(5):753. doi: 10.3390/genes12050753 (PMC8156360; doi:10.3390/genes12050753)

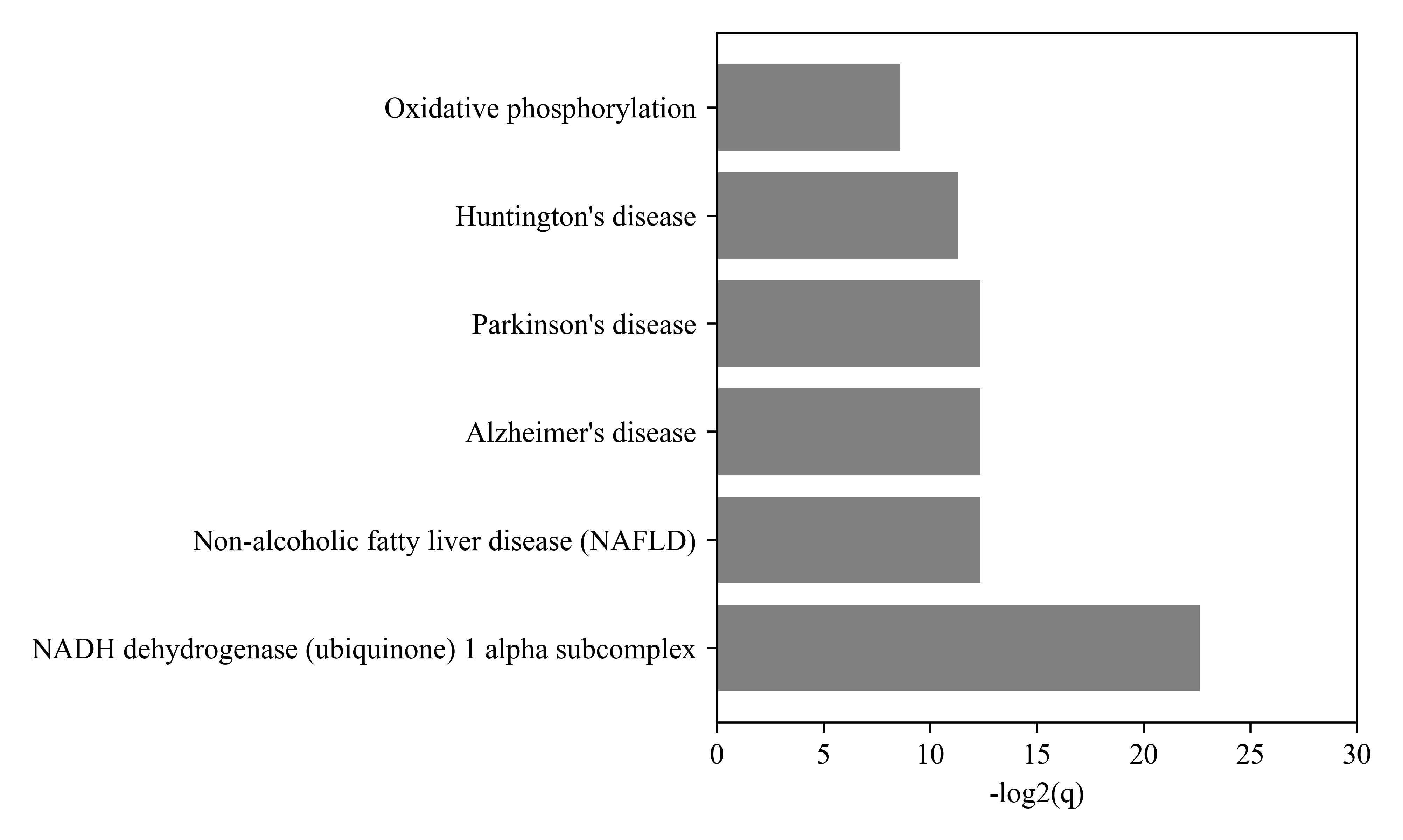

Supplement: Supplementary file 1 [file genes-12-00753-s001.zip › supplementary file/FIG. S1.tif]

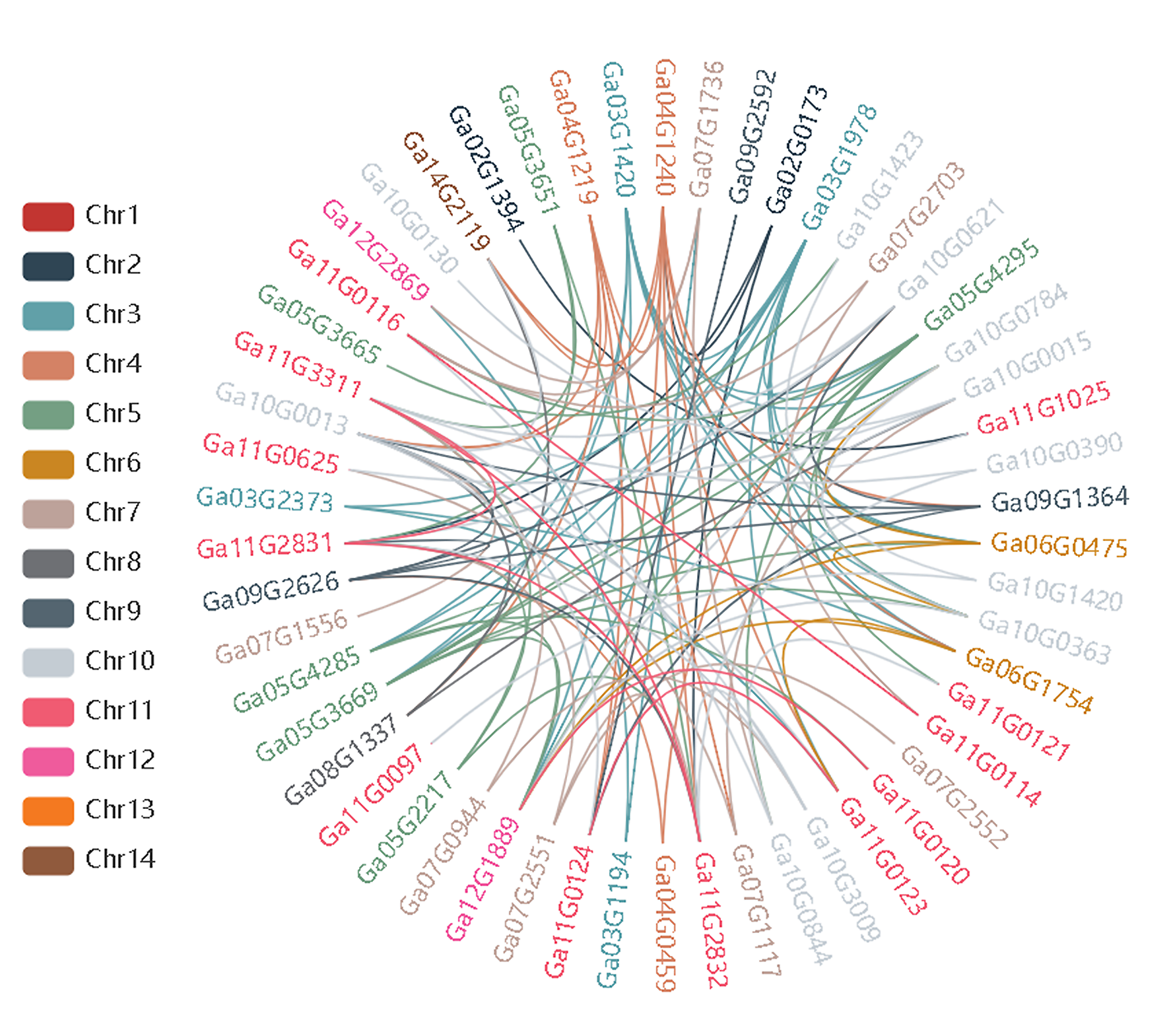

Supplement: Supplementary file 1 [file genes-12-00753-s001.zip › supplementary file/FIG. S2.tif]

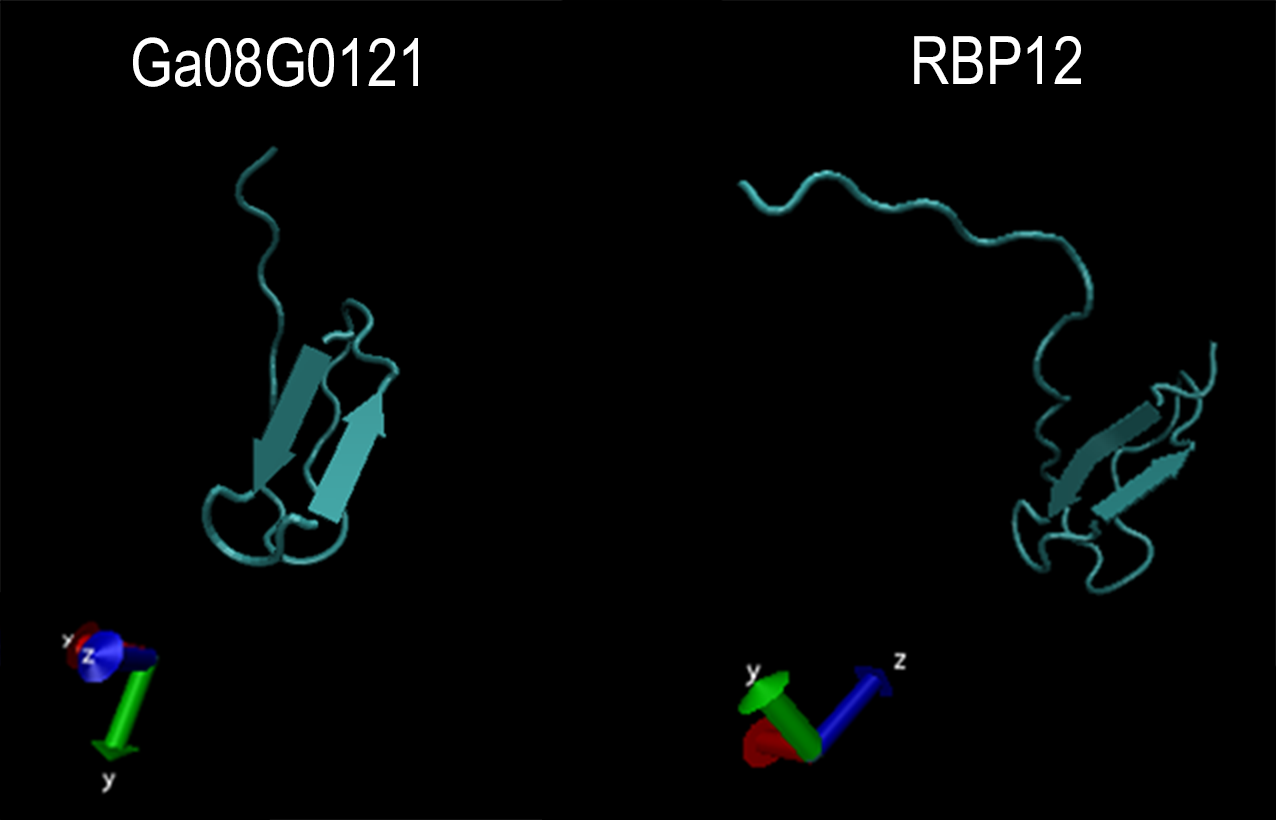

Supplement: Supplementary file 1 [file genes-12-00753-s001.zip › supplementary file/FIG. S3.tif]
